# The Endocrine Function of Osteocalcin Regulated by Bone Resorption: A Lesson from Reduced and Increased Bone Mass Diseases

**DOI:** 10.3390/ijms20184502

**Published:** 2019-09-11

**Authors:** Michela Rossi, Giulia Battafarano, Jessica Pepe, Salvatore Minisola, Andrea Del Fattore

**Affiliations:** 1 Bone Physiopathology Group, Multifactorial Disease and Complex Phenotype Research Area, Bambino Gesù Children’s Hospital, IRCCS, 00146 Rome, Italy; michela_r10@yahoo.it (M.R.); giulia.battafarano@opbg.net (G.B.); 2 Department of Internal Medicine and Medical Disciplines, Sapienza University of Rome, 00186 Rome, Italy; jessica.pepe@uniroma1.it (J.P.); salvatore.minisola@uniroma1.it (S.M.)

**Keywords:** osteocalcin, osteoclast, bone diseases

## Abstract

Bone is a peculiar tissue subjected to a continuous process of self-renewal essential to assure the integrity of the skeleton and to explicate the endocrine functions. The study of bone diseases characterized by increased or reduced bone mass due to osteoclast alterations has been essential to understand the great role played by osteocalcin in the endocrine functions of the skeleton. The ability of osteoclasts to regulate the decarboxylation of osteocalcin and to control glucose metabolism, male fertility, and cognitive functions was demonstrated by the use of animal models. In this review we described how diseases characterized by defective and increased bone resorption activity, as osteopetrosis and osteoporosis, were essential to understand the involvement of bone tissue in whole body physiology. To translate this knowledge into humans, recently published reports on patients were described, but further studies should be performed to confirm this complex hormonal regulation in humans.

## 1. Introduction

Bone is a very active tissue subjected to a continuous process of remodeling by which ischemic and microfractured bone is replaced by newly mechanical competent bone. The bone remodeling activity is also essential to regulate calcium and phosphate homeostasis. In recent years, it has been well established that the skeleton represents an endocrine organ, able to regulate energetic metabolism [1], insulin secretion [2,3], male fertility [4], muscle activity [5,6], cognitive functions, and behavior [7].

Bone remodeling is regulated by the concerted action of osteoblasts for bone formation and osteoclasts involved in bone resorption. Osteoclasts are giant multinucleated cells derived from the fusion of mononuclear precursors belonging to the monocyte/macrophage lineage [8]. Their activity to resorb bone is an essential step of the remodeling cycle, where they collaborate closely with osteoblasts in the basic multicellular unit [9,10,11]. The bone resorption activity consists of two sequential steps: the acidification of resorbing lacuna to dissolve the mineral component hydroxyapatite (HA) and the secretion of proteolytic enzymes to digest the organic part of the bone matrix. For the dissolution of hydroxyapatite crystals, an acidic pH (pH~4.5) in the lacuna is required. Carbonic anhydrase II (CAII) hydrates carbonic anhydride (CO_2_) to produce carbonic acid (H_2_CO_3_) that spontaneously dissociates in bicarbonates (HCO_3_^–^) and protons (H^+^). Hydrogen ions are vehicled in the resorption lacuna by the vacuolar proton pump V-H^+^ATPase [12], while HCO_3_^–^ is switched with chloride (Cl^–^) by a HCO_3_^–^/Cl^–^ exchanger situated in the basolateral domain. Cl^–^ ion is transported in the resorption lacuna by a Cl^–^/H^+^ antiport, balancing the charge of ions across the membrane [13,14]. Dissolution of mineral crystals exposes the collagen-rich matrix to proteolytic enzymes, principally the acidic hydrolases such as cathepsin K, and matrix metalloproteinases (MMPs), including MMP9, which are released by osteoclasts into the resorption lacuna [15].

To maintain the integrity of the skeleton, bone remodeling needs to be perfectly balanced with a resorption phase followed by bone formation. When imbalance between bone resorption and formation occurs, skeletal abnormalities are observed, leading to the onset of several bone diseases including bone loss diseases i.e., osteoporosis, and increased bone mass disorders, such as osteopetrosis and pycnodysostosis [16]. In this review we describe how the study of bone resorption-related diseases has been important to understand the role of osteoclasts and bone resorption in the regulation of the endocrine functions of the skeleton.

## 2. Disorders of Altered Bone Resorption

Physiological bone remodeling is a multistep process that can be altered by various factors, including hormonal changes, age-related factors, drugs, and secondary diseases, leading to the onset of bone-related disorders both in women and men.

Osteoporosis is a common disorder of bone remodeling characterized by reduced bone mass and qualitative alterations of bone tissue, leading to increased risk of fractures [17]. It is an asymptomatic condition until the first fracture occurs. The fractures are associated with substantial pain and suffering, disability, and even death, along with substantial costs to society. Primary osteoporosis is associated with estrogen deficiency in postmenopausal women. Moreover, several diseases and drugs may cause secondary osteoporosis, such as glucocorticoid treatment, prolonged immobility, and tumors [18]. The pathogenesis of osteoporosis is complex and caused by increased bone turnover that leads to continuous and progressive bone loss [19]. The goal of pharmacological therapy is to inhibit the excessive bone resorption and enhance bone formation, in order to reduce the risk of fractures. Approved therapies are based on antiresorptive drugs, such as bisphosphonates or the anti-RANKL (receptor activator of nuclear factor kappa-Β ligand) antibody denosumab, which help to preserve the existing bone mass and increase the degree and homogeneity of mineralization; or anabolic agents, such as teriparatide, abaloaratide and romosozumab, which stimulate bone formation [20,21,22].

Diseases associated with reduced bone resorption are less common and often display a genetic basis. Osteopetrosis, firstly described in 1904 and called “marble bone disease” [23], represents a group of heterogeneous, rare, genetic diseases characterized by increased bone mass, with an incidence of up to 1:100,000, caused by the failure of bone resorption by osteoclasts. Three different types of osteopetrosis are classified in humans, characterized by different way of inheritance and severity, from asymptomatic to fatal [24]. In Table 1 the genes mutated in osteopetrotic patients are listed.

Autosomal recessive osteopetrosis (ARO) is a particularly severe form of the disease due to bone resorption defects because of osteoclast absence or dysfunction. Patients have increased bone mineral density (BMD), ‘bone in bone’ appearance, growth retardation, eye protrusion, macrocephaly, hydrocephaly, frontal bossing, deafness, and blindness due to cranial nerve compression, severe anemia, pancytopenia, and hepatosplenomegaly. Two different forms of the disease can be distinguished by histological analysis: the osteoclast-rich, characterized by high number of osteoclasts, and the osteoclast-poor where no osteoclasts are observed in bone biopsies [24,25,26,27,28]. Intermediate recessive osteopetrosis (IRO) is milder than ARO and it is characterized by short stature, bone sclerosis, pathological fractures, dental malformations, and jaw osteomyelitis. Autosomal dominant osteopetrosis (ADO) is the adult form of osteopetrosis. Patients usually display thickness of the skull base, vertebral end plates (sandwich vertebrae or Rugger–Jersey spine) and pelvis, and spontaneous fractures. ADO is characterized by a heterogeneous range of clinical presentations, from asymptomatic to very severe, and early death is rare [29,30]. Seventy percent of the cases are explained by mutations of the *CLCN7* gene [25].

Another disease with increased bone mass is pycnodysostosis with some features similar to osteopetrosis. However, the affected individuals have characteristic facies, beaked nose, blue sclera, short stature, aplasia of the digits, and increase of bone mass, although not sufficient to obliterate medullary canals [31,32]. Pycnodysostosis is an autosomal recessive disorder due to loss-of-function mutations of the *CTSK* gene encoding the cysteine protease cathepsin K, which is responsible for degradation of collagen type I and other bone proteins. Cathepsin K deficient osteoclasts can dissolve the inorganic bone matrix but cannot degrade the organic part [33].

## 3. Osteocalcin

The relevance of bone resorption studies for the endocrine functions of the skeleton has been demonstrated starting from the first paper showing the regulation of insulin secretion by osteocalcin [3].

Osteocalcin (Ocn or bone γ-carboxyglutamic acid (Gla) protein, BGP) is a small (49 amino acids in humans) non-collagenous protein secreted by osteoblasts and partially stored in the bone matrix [34,35]. The protein was first isolated by Price et al. [36,37] from bovine and human bone and it is the most abundant of the Gla-containing proteins in bone. For many years, osteocalcin was described as a marker of bone formation and it was believed to regulate mineralization; however, this protein has many features resembling a hormone. Osteocalcin is produced by osteoblasts as a pro-peptide that is cleaved before its secretion to remove an endoplasmic reticulum signal sequence and the pro-sequence [38]. In the circulation its concentration is ng/mL and its levels are regulated by a circadian rhythm. In humans osteocalcin levels are very low in the morning, they started to raise in the afternoon, and reach a peak in the night [39].

**Figure 1:**
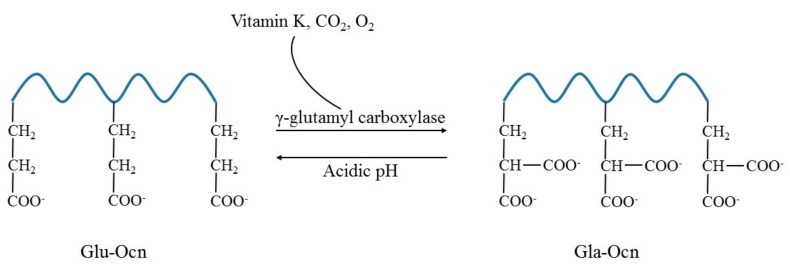
Representation of osteocalcin post-translational modification. Post-translational carboxylation at three glutamic acid residues occurs by γ-glutamyl carboxylase that uses vitamin K, CO_2_, and O_2_ as cofactors. The carboxylated form of osteocalcin (Gla–Ocn) can be converted into a form with a lower grade of carboxylation (Glu–Ocn) by acidic pH.

Osteocalcin contains three glutamate residues that can be γ-carboxylated; this modification allows its binding to calcium and hydroxyapatite (Figure 1). This modification is catalyzed by a γ-glutamyl carboxylase that utilizes vitamin K, CO_2_, and O_2_ as cofactors, supplied by the vitamin K cycle and circulation [34,40]. The osteocalcin with a reduced degree of carboxylation on three glutamate residues (undercarboxylated osteocalcin, Glu–Ocn) is available with less affinity for hydroxyapatite and easily released to the circulation [41,42,43] (Figure 1).

Three decades ago, two observations revealed a complex regulation of osteocalcin structure and release from the bone matrix: 1. Carboxylated osteocalcin (Gla–Ocn) bound to the mineralized bone matrix via its Gla residues can be released upon resorption by osteoclasts [37,41,44,45]; 2. The decarboxylation of proteins is a process that can be stimulated by acid pH (Figure 1) [45,46].

**Figure 2:**
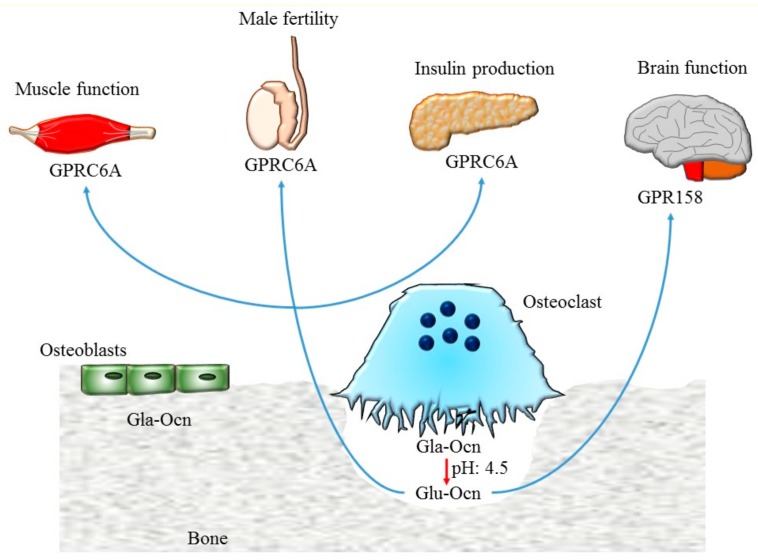
Schematic representation of osteocalcin functions. Osteocalcin stored in the bone matrix in the carboxylated form (Gla–Ocn) is decarboxylated by acidic pH in the resorption lacuna. The undercarboxylated osteocalcin (Glu–Ocn) is released into the circulation and regulates muscle function, male fertility, and insulin secretion by its binding to the GPRC6A receptor while it controls cognitive functions through the GPR158 receptor.

These notions led to investigate the role of osteoclasts for osteocalcin modification. The first evidence was given from the paper of Ferron et al. [3] that demonstrated how bone resorption by osteoclasts is essential for the undercarboxylation of osteocalcin stored in the bone matrix, and thus released in the resorption lacuna (Figure 2).

Indeed, the ratio of undercarboxylated and carboxylated osteocalcin was significantly increased when osteocalcin was exposed to pH 4.5. Interestingly, this value of pH was observed in the resorption lacuna created by osteoclasts. To demonstrate that osteoclast resorption is essential for the activation of osteocalcin, in vitro experiments were performed. Osteoclast precursors were plated on bovine cortical bone and differentiated by treatment with the osteoclastogenic cytokine RANKL. The measurement of the total, carboxylated, and undercarboxylated osteocalcin revealed an increase of the Glu–Ocn form and a reduction of Gla–Ocn, leading to a 2-fold increase in the Glu/Gla ratio [3]. The relevance of osteoclast activity in the regulation of osteocalcin was further confirmed by the observation of the osteopetrotic *oc/oc* mice carrying loss-of-function mutation of *tcirg1* (T cell immune regulator 1) gene encoding α3 subunit V-H^+^ATPase; this animal was characterized by defective acidification of lacuna and impaired bone resorption. The undercarboxylated osteocalcin in *oc/oc* was 30% of levels revealed in wild type (WT) serum. To demonstrate that this alteration was secondary to the dysfunction of Tcirg1 in osteoclasts, *oc/oc* fetal liver hematopoietic stem cells were transplanted into WT irradiated mice. A high bone mass phenotype was observed in WT recipient mice for the inability of osteoclasts to resorb bone. Interestingly, no alterations were revealed in the total levels of osteocalcin, while a reduction of the serum undercarboxylated form was observed. Finally, a reduction of the active form of osteocalcin was measured in six patients affected by autosomal dominant osteopetrosis, characterized by defective acidification ability of osteoclasts to acidify, as evidenced by assay with LysoSensor fluorescent pH indicators [3].

Up to now two different receptors were identified for osteocalcin belonging to the G protein-coupled receptor family C: GPRC6A and GPR158. In human, chimpanzee, and small species GPRC6A is expressed in hepatocytes, pancreatic β-cells, Leydig cells, skeletal muscle myocytes, kidney proximal/distal tubules, and placenta. At least two signaling pathways were induced after the binding of osteocalcin to the receptor: 1. the IP3 (inositol 1, 4, 5-trisphosphate)-Ca^2+^ pathway activated by the action of phospholipase C (PLC); 2. the adenylyl cyclase-cAMP-PKA (protein kinase A) pathway that leads to the activation of the MEK (mitogen-activated protein kinase)–ERK (extracellular signal regulated kinase) cascade [47,48]. Three isoforms for the receptor with differential expression were known (1365, 853, and 1165 bp). Isoform 1 is expressed in many tissues including pancreas, testis, brain, liver, kidney, placenta, and skeletal muscle while the other two isoforms are less abundant [47,49]. The binding of undercarboxylated osteocalcin to this receptor stimulates the production and the release of insulin from pancreatic beta cells and enhances testosterone synthesis in Leydig cells [50].

GPR158 receptor is expressed in the cortex, hippocampus, midbrain, brainstem, and cerebellum. The binding of osteocalcin to GPR158 stimulates the histone-binding protein RbAp48, which in turn regulates GPR158 and BDNF (brain-derived neurotrophic factor) [51,52]. Interestingly, reduction of RbAp48 expression is a molecular hallmark of memory loss correlated with aging in humans and mice [53]. It was demonstrated that osteocalcin/GPR158/RbAp48 signaling acts on the dental gyrus/CA3c and CA3a brain area to modulate fear discrimination and completion, respectively. The activation of this pathway in aged subjects alleviates the cognitive impairment associated with aging [52].

## 4. Glucose Metabolism

The *oc/oc* mice are characterized by reduced serum level of undercarboxylated osteocalcin, reduced levels of insulin, and glucose intolerance. To confirm that this phenotype was due to defective osteoclast function, Ferron et al. transplanted *oc/oc* fetal liver hematopoietic stem cells into WT irradiated mice; an increase of bone mass, and reduced levels of undercarboxylated osteocalcin and insulin with an increase of glucose levels strengthened the concept that bone resorption is essential for the regulation of glucose metabolism [3].

Indeed, ADO patients showed decreased levels of undercarboxylated osteocalcin and insulin [3]. These data were important to demonstrate that osteocalcin with a reduced level of carboxylation is relevant for the regulation of whole-body glucose metabolism.

Moreover, mice with a deletion of RANKL decoy receptor osteoprotegerin (Opg) were characterized by increased bone resorption activity and the osteoporosis phenotype due to loss of the inhibitory signal for osteoclasts; these animals displayed improved glucose tolerance and enhanced insulin sensitivity. These results clearly showed that bone resorption is beneficial for glucose metabolism, and increased bone resorption activity should be associated with better glucose control [3].

The notion that bone resorption is tightly linked to glucose homeostasis is clinically important, since most of the drugs used in the treatment of bone disease patients target this aspect of bone remodeling. Specifically, high levels of fasting plasma glucose are measured in osteoporotic women treated with bone resorption inhibitory drugs, and there is a significant and positive correlation between undercarboxylated Ocn and urinary cross-linked N-telopeptides of type I collagen (NTX) [54,55].

Moreover, the use of anti-resorptive drugs should increase the risk of insulin resistance and diabetes due to reduced levels of undercarboxylated osteocalcin. However, epidemiological and clinical trials concluded that the use of anti-resorptive drugs was not associated with alterations of plasma glucose, insulin resistance, and diabetes development, but was associated with a decreased risk of diabetes, especially for long-term treatments [56,57,58,59,60,61].

In a recently published paper, Urano et al. [62] performed a study including 1691 Japanese postmenopausal women; 371 subjects were treated with bisphosphonates. Serum osteocalcin levels were significantly correlated with HbA1c levels among postmenopausal women. Moreover, they showed that a decrease (<6.1 ng/mL) of serum osteocalcin was associated with future development of type 2 diabetes mellitus. However, the authors concluded that there was no trend of increase in incident diabetes rate in bisphosphonate-treated patients [62]. Further studies should be performed to unravel the association between bisphosphonate treatment and type 2 diabetes mellitus onset.

## 5. Male Fertility

Since osteocalcin knock-out mice showed reduced male fertility due to Leydig cell hypoplasia and hypotestosteronemia, Karsenty’s group demonstrated that the undercarboxylated osteocalcin is able to bind its receptor GPRC6A expressed by Leydig cells and stimulate the testosterone synthesis [4,63]. To evaluate the relevance of the bone resorption activity in the regulation of male fertility, Ctsk–Cre;DTA^fl/+^ mice were generated. These animals derived from the crossing of Ctsk^Cre/+^ mice, which express Cre recombinase under the control of the cathepsin K locus, with diphtheria toxin A (DTA)^fl/+^ mice that express a flox–stop–flox DTA cassette under the control of the Rosa26 locus. Cre-mediated removal of the stop cassette in mature osteoclasts leads to the selective death of these cells. This animal model was characterized by very dense bones and the absence of incisor eruption due to severe impairment of bone resorption, and died at 2–3 weeks of age. Due to premature death of mice, the Ctsk–Cre;DTA^fl/+^ fetal liver hematopoietic stem cells were transplanted into WT animals. An increase of bone mass with high number of osteoclasts was observed in the transplanted animals. Moreover, a reduction of undercarboxylated osteocalcin levels was revealed in these animals with a parallel decrease of sperm count, testis weight, testosterone levels, and expression of *StAR*, *Cyp11a*, *Cyp17*, and *3β-HSD* genes encoding enzymes required for testosterone biosynthesis. These defects related to male fertility were corrected after treatment for 30 days with recombinant osteocalcin [4].

The osteocalcin-mediated regulation of male fertility was further confirmed by osteoporotic *opg–/–* mice that presented a huge increase in osteoclast number [4]. In this mouse model, the serum level of active osteocalcin was increased with the testosterone level; in addition, testes, seminal vesicles, and epididymal weights, and the sperm count were also increased. Thus, these mouse models showed the importance of bone resorption in the regulation of male fertility mediated by active osteocalcin [4].

The association between osteocalcin and male fertility has also been investigated in human. A positive association was revealed between osteocalcin and testosterone serum concentrations along with serum C-terminal telopeptides of type I collagen (CTX) [64].

Furthermore, in a different study on type 2 diabetic patients, the levels of circulating undercarboxylated osteocalcin positively correlated with testosterone, confirming the direct action of osteocalcin on testosterone production [65]. These results are in contrast with a previous report showing no associations between testosterone serum levels, osteocalcin, and CTX in 40 middle-aged healthy men and 80 osteoporotic patients [66]. Moreover, Liu et al. performed a meta-analysis to compare the osteocalcin with testosterone concentrations in primary osteoporotic males and age matched controls. No significant alterations were observed between serum total Ocn and testosterone levels. However, there were some limitations of this study concerning the low number of case-control studies (*n* = 5) and the evaluation of the total osteocalcin instead of undercarboxylated form [67]. Therefore, well-designed studies should be performed for a better understanding of the complex regulation between osteocalcin, bone resorption, and male fertility.

## 6. Brain Functions

An unexpected peripheral effect of osteocalcin is its central role in the regulation of cognitive function. In an elegant paper published by Oury et al., it was demonstrated that circulating osteocalcin is able to cross the blood–brain barrier and to bind the GPR158 receptor, regulating cognitive functions and anxiety-related behavior [7].

Recent papers revealed an association between osteoporosis and cognitive impairment and dementia [68,69]. In the Framingham study, Tan et al. found that lower BMD was associated with the risk of developing Alzheimer’s disease [70]. Moreover, in a longitudinal study, 64 participants, aged 34–87 years old (62.78 ± 9.27), were recruited and cognitive and clinical examinations were registered at baseline and after 3 years. A significant association between BMD and lean body mass and memory abilities was revealed [71]. Structural analysis of bone health and neuropsychological functions suggested that lower BMD is associated with lower cognitive performance, particularly in post-menopausal women. Zhou et al. showed relationships between BMD and Alzheimer’s disease and cognitive decline, particularly in older women [72]. Furthermore, a recent study on obese and control subjects found that lower Ocn was associated both with higher body mass index (BMI) and with worse cognitive performance and brain microstructural changes [73]. Puig et al. calculated that serum Ocn independently explained 10% of the variation in cognitive performance [73]. A cross-sectional study including 225 elderly (52% women, age: 74.4 ± 3.3 years) and 134 young (52% women, age: 23.4 ± 2.7 years) participants revealed that osteocalcin levels were positively associated with measures of executive functioning and global cognition in the older women [74].

Further longitudinal studies investigating bone remodeling (as well as BMD) and cognitive performance in older women are needed to elucidate the relationship between bone health and neuromodulation.

## 7. Conclusions

Bone was considered for long time to be an inert structure necessary for calcium homeostasis, mobility, and maintenance of the hematopoietic niche. The pioneering studies of the Karsenty group on osteocalcin as a hormone revealed a more complex role of the bone as regulator of the whole organism. However, many of the functions associated with osteocalcin still need to be confirmed in humans [75]. First of all, it should be considered that murine and human osteocalcin show a different pattern of carboxylation. Osteocalcin is γ-carboxylated on the glutamic acids (GLU) at positions 13, 17, and 20 of protein in mouse, and on GLU 17, 21, and 24 in humans [34]. Moreover, the possibility to use Glu–Ocn as a prognostic or pathogenic marker for metabolic–endocrine disorders remains to be confirmed since no universal standardized method exists for its measurements; osteocalcin should be evaluated by HA binding assay, by electro-chemiluminiscence immunoassay, or by ELISA.

The HA-based measurement of osteocalcin is based on the lower binding affinity for HA of the undercarboxylated osteocalcin compared to the carboxylated form. This binding assay uses HA to bind and to sequester carboxylated osteocalcin; the resulting supernatant is used to measure the undercarboxylated Ocn by immunoassay [76,77].

Such analysis of undercarboxylated osteocalcin represents a semi-quantitative method that does not precisely quantify the serum concentration of undercarboxylated or carboxylated forms. Therefore, measurement of Glu–Ocn is not straightforward and the available methods do not discriminate the number and position of uncarboxylated Glu residues; these limitations must be considered in the interpretation of results. Moreover, the circadian alterations of osteocalcin differ, and should be evaluated, between mice and humans. In mice, Ocn levels reach a peak during the light period and are very low during the dark hours, whereas in humans, the levels fall in the early morning and rise in the afternoon with a peak at night [39]. These changes may be associated with modulation of glucocorticoid levels. Heshmati et al. showed that in humans, the rise of serum cortisol in the morning is responsible for the daytime nadir of serum osteocalcin levels and that the nocturnal increase of osteocalcin, then, is a consequence of the declining cortisol levels in the evening and nighttime hours [78]. In rodents, corticosterone levels are constant from early morning to afternoon; a significant elevation is revealed at 20:00 in the evening [79]. Therefore, the time point at which samples are collected should be taken into account for the extrapolation of results from mouse studies into the human setting.

However, the studies published in the last 10 years have hugely revolutionized the knowledge about bone tissue, making the skeleton a survivor organ during evolution. Indeed, bone tissue made possible the transition of life from water to land, from an external calcium rich environment to a habitat where it was necessary to have an internal reservoir of this ion; since it cooperates with muscles in the regulation of walking and running, bone enabled animals to escape danger and to find food. At the same time, through its endocrine functions, bone may have supported the survival in hostile environments. This evolutionary theory could explain the reason for the central role of the skeleton in the regulation of the whole body physiology. The study of bone diseases characterized by increased or reduced bone mass is very important to understanding the endocrine functions of the skeleton and will allow identification of new roles of bone in inter-organ communication.

## Figures and Tables

**Table 1:** List of genes mutated in osteopetrotic patients.

Osteopetrosis	Genetic Transmission	Gene Mutation	Protein
ARO	Autosomal recessive osteopetrosis	*TCIRG1*	α3 subunit V-H^+^ATPase
		*CLCN7*	Chloride channel 7
		*OSTM1*	Osteopetrosis associated transmembrane protein
		*PLEKHM1*	Pleckstrin homology domain containing family M, member I
		*SNX10*	Sorting nexin 10
		*TNFSF11*	Receptor activator for nuclear factor *κ*B ligand
		*TNFRSF11A*	Receptor activator for nuclear factor *κ*B
IRO	Autosomal recessive osteopetrosis	*CAII*	Carbonic anhydrase
ADO	Autosomal dominant osteopetrosis	*CLCN7*	Chloride channel 7
